# Autologous Osteochondral Transplantation in Large Osteochondral Defects—A Follow-Up of 52 Patients After Knee Joint Resurfacing

**DOI:** 10.3390/jcm14176180

**Published:** 2025-09-01

**Authors:** Alice Wittig-Draenert, Martin Breitwieser, Jörn Wittig, Jürgen Bruns

**Affiliations:** 1 Department for Orthopedic Surgery and Traumatology, Paracelsus Medical University, 5020 Salzburg, Austria; m.breitwieser@salk.at; 2 Department for Oral and Maxillofacial Surgery, Paracelsus Medical University, 5020 Salzburg, Austria; 3 Department for Orthopedic Surgery, Diaconial Hospital Hamburg, 22767 Hamburg, Germany

**Keywords:** autologous osteochondral transplantation (AOT), cartilage repair, knee joint resurfacing, osteochondral defect (OD), donor site morbidity, surgical diamond techniques, diamond bone cutting system (DBCS)

## Abstract

**Background:** Autologous osteochondral transplantation (AOT)—the transfer of hyaline cartilage with its underlying subchondral bone—is well established for focal osteochondral lesions, yet evidence for larger (>200 mm^2^) defects is limited. We assessed clinical and functional outcomes of AOT in patients with osteochondral knee lesions exceeding 200 mm^2^. **Methods:** In this retrospective cohort study, 52 patients underwent AOT for full-thickness osteochondral defects of the femoral condyles or patellofemoral joint. All lesions were ≥200 mm^2^ and treated with a standardized press-fit technique using one to four overlapping cylindrical grafts. Pain and knee function were evaluated preoperatively and at 3, 6, and 12 months postoperatively with the Visual Analogue Scale (VAS), Tegner–Lysholm Knee Score (TLKS), and Knee Society Score (KSS). **Results:** Mean defect size was 224.4 ± 84.5 mm^2^. The VAS improved from 6.32 ± 1.1 preoperatively to 0.72 ± 0.6 at 12 months (*p* < 0.001). The TLKS rose from 58.6 ± 11.4 to 95.0 ± 6.8 and the KSS from 63.8 ± 12.2 to 97.4 ± 4.9 during the same period (both *p* < 0.001). Most gains occurred within the first 3–6 months and were sustained at 12 months. No major surgical complications were observed, and outcomes were unaffected by age, sex, or graft number/size. **Conclusions:** AOT is a safe, effective option for large osteochondral knee defects (>200 mm^2^), offering rapid, durable pain relief and excellent functional recovery while preserving native joint structures. Accurate donor site reconstruction and precise graft placement in the weight-bearing zone appear critical for optimal results. Longer-term prospective studies are needed to confirm durability and refine patient-selection criteria.

## 1. Introduction

Cartilage damage in the knee joint is a common and often serious issue in orthopedic surgery. If left untreated, these injuries can lead to larger defects, chronic pain, and early osteoarthritis [1,2,3]. Among the surgical options available, autologous osteochondral transplantation (AOT) stands out as a promising technique—especially for full-thickness lesions that reach into the underlying bone [4,5]. AOT allows for the transfer of living cartilage along with its supporting bone, which helps restore the joint’s natural structure and function [6,7].

Unlike cartilage-only strategies whose efficacy may decline as defect size increases, AOT restores the osteochondral unit—native hyaline cartilage plus subchondral bone—providing immediate mechanical stability and physiological load transmission, which is particularly relevant in lesions ≥ 200 mm^2^ in size. For smaller or medium-sized defects, AOT has shown reliable and encouraging outcomes [8,9,10]. However, treating larger areas or reconstructing entire joint surfaces is more difficult [11]. Reports on these cases have shown mixed results, often influenced by the complexity of the procedure and differences in surgical approach [12,13].

**Figure 1:**
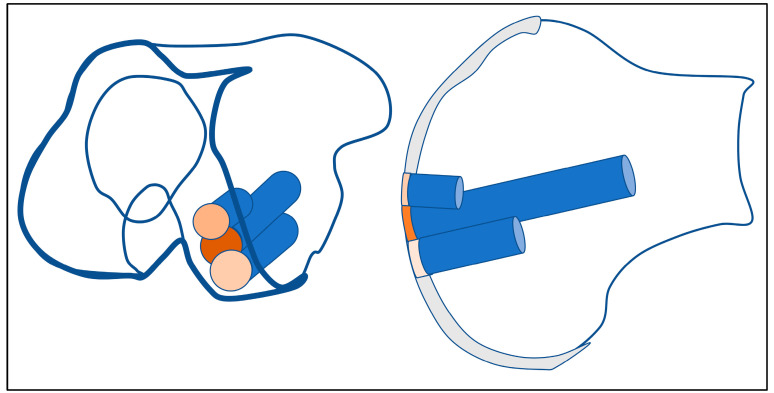
Mastercard or snowman sign—triple-graft in the weight-bearing joint surface of the medial knee condyle. For stability reasons, it is important to have the first implanted graft long and deep in the middle space (dark red), and the other grafts slightly overlapping and shorter, so the deep edge is supported by the first cylinder, and protrusion of the second and third graft is blocked.

A key factor in the success of these procedures is how well the transplanted bone and cartilage integrate with the surrounding tissue. This becomes especially important when more than one cylindrical graft is used [14]. For larger defects, the precise alignment of overlapping grafts is crucial to ensure stability and avoid problems like graft shifting under pressure. Some experts suggest starting with a longer central graft and surrounding it with slightly shorter ones to create a stable fit—often described by other authors as a “snowman” or “Mastercard” configuration [15,16,17] (Figure 1).

It is also important to look beyond the recipient site. Donor sites, usually located in the same knee, can also cause problems if not treated carefully [18,19,20]. In many cases, these areas are left unfilled, which can lead to bleeding, pain, or long-term healing issues. Studies show that filling donor sites with bone grafts or substitutes can reduce these risks and help preserve joint function [21,22,23,24].

Although AOT is well established for smaller lesions, research specifically addressing its effectiveness in treating large osteochondral defects, especially those requiring multiple overlapping grafts, remains scarce [17]. With this retrospective study, we aim to help fill that gap by reporting on the short- to medium-term outcomes of 52 patients who underwent knee reconstruction using the AOT technique with a minimum lesion size of 200 mm^2^. Our goal is to better understand how this approach performs in real-world clinical practice.

## 2. Materials and Methods

### 2.1. Study Design and Setting

This investigation was designed as a retrospective cohort study evaluating the clinical and functional outcomes following autologous osteochondral transplantation (AOT) in patients with large osteochondral knee defects. This study includes data from patients treated between November 2009 and November 2014 at a tertiary orthopedic center in Hamburg, Germany. Anonymized data analysis and statistical evaluation were subsequently conducted at the Department of Orthopedics and Traumatology, University Hospital Salzburg, Austria.

### 2.2. Patient Selection

Patients were eligible if they were ≥14 years old and had focal osteochondral knee defects (International Cartilage Repair Society [ICRS] grade III–IV) of the femoral condyles or patellofemoral joint with a minimum lesion size of 200 mm^2^. Throughout this manuscript, we use the following terminology: a full-thickness cartilage lesion denotes ICRS grade III–IV damage confined to cartilage, whereas an osteochondral defect denotes cartilage loss with concomitant loss or compromise of the subchondral bone. The present cohort comprised osteochondral defects as defined above. All patients were treated with autologous osteochondral transplantation (AOT) using a standardized press-fit technique and completed ≥3 months of postoperative follow-up.

Exclusion criteria included the following:

- Incomplete surgical procedures;
- Re-injury of the operated joint during follow-up;
- Incomplete or missing clinical data.

A total of 52 patients were included in the final analysis after applying these criteria. Baseline demographic data (age and sex) and clinical characteristics (defect location and size) were extracted for all included patients.

### 2.3. Data Collection

Data were retrospectively obtained from electronic medical records, surgical reports, and follow-up documentation. Standardized follow-up intervals were scheduled at 3 months, 6 months, and 12 months postoperatively. Clinical scores were documented by the operating surgeon or a designated clinician during routine follow-up. Missing data were addressed through exclusion from individual score analyses. As only one surgeon and team conducted the procedures and evaluations, inter-rater reliability was not applicable.

### 2.4. Surgical Technique

**Figure 2:**
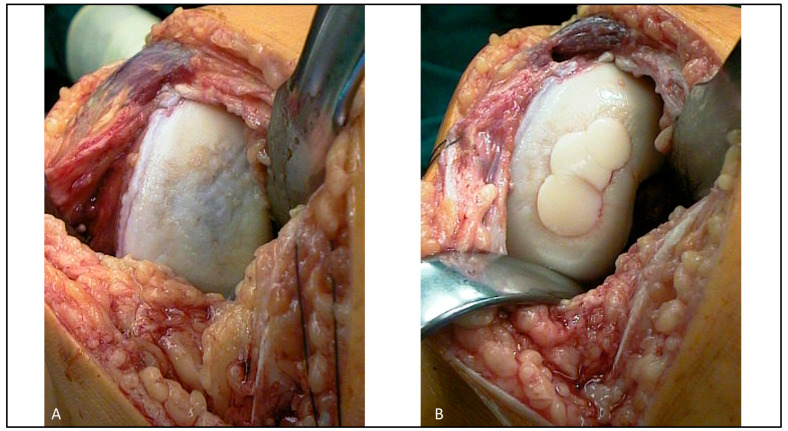
Mastercard or snowman sign—intraoperative photograph before (**A**) and after (**B**) triple-graft transplantation of a 33-year-old male with severe post-traumatic destruction of the hyaline surface in the weight-bearing zone of the medial knee compartment.

All procedures were performed by an experienced orthopedic surgeon specializing in cartilage reconstruction. Osteochondral cylindrical plugs (diameter: 11.6–14.1 mm) were harvested using a water-cooled diamond cutting system from non-weight-bearing areas of the ipsilateral knee, specifically the lateral or medial wing of the patellar groove. The plugs were transplanted into the defect zone in a press-fit technique, forming mono-, double-, or triple-overlapping configurations depending on lesion size and location (Figure 1 and Figure 2).

Donor site management was standardized in all cases. The harvest site in the patellofemoral groove was reconstructed using press-fit autologous bone cylinders obtained from the iliac crest, each covered with musculoskeletal fibers to promote integration and healing. The defect at the iliac crest itself was filled with a press-fit hydroxyapatite-based bone substitute, and the musculotendinous sheath was closed over the site to ensure stability and minimize postoperative morbidity.

Perioperative care included standardized rehabilitation protocols with partial weight bearing for up to six weeks. The surgical technique emphasized precise congruency between the graft and the recipient site to optimize mechanical stability and graft integration [13,25].

### 2.5. Outcome Measures

Clinical outcomes were assessed using the following validated scores:

- The Visual Analog Scale (VAS) for pain;
- The Tegner–Lysholm Score (TLS) for knee function;
- The Knee Society Score (KSS) for functional assessment;
- The Modified Cincinnati Score (MCS) for patient-reported knee function.

These scores were recorded preoperatively and postoperatively at 3, 6, and 12 months. The primary endpoint was the change in pain and functional scores over time. Secondary endpoints included subgroup analyses based on graft configuration (mono-, double-, and triple-cylinder chains), patient sex, patient age, and defect size.

### 2.6. Statistical Analysis

Descriptive statistics were used to summarize baseline characteristics and outcome data. Generalized Estimation Equation (GEE) models were employed to evaluate longitudinal changes, testing data for normality and assessing key predictors including age, sex, defect size, treatment site, and time. Post hoc analyses were performed using Least Significant Difference (LSD) testing. Spearman correlation coefficients were computed for continuous variable associations. Confidence intervals (95% CI) were visualized using whisker plots.

All analyses were conducted using IBM SPSS Statistics for Windows, Version 29.0.2.0 (IBM Corp., Armonk, NY, USA), and TIBCO Spotfire, Data Science Workbench Version 14. A *p*-value of < 0.05 was considered statistically significant.

### 2.7. Quality Assessment

To reduce potential bias, data were extracted and verified by the principal investigators. Only complete clinical records with consistent follow-up were included in the final analysis. Complications were classified according to severity and relevance, including donor site morbidity, infection, bleeding, and nerve injury. No major intraoperative or postoperative complications occurred in the analyzed cohort.

### 2.8. Ethics

This study was approved by the Ethics Committee for the State of Salzburg (Austria). Given the retrospective and fully anonymized nature of the dataset, a waiver of informed consent was granted. All procedures adhered to the principles of the Declaration of Helsinki and national ethical standards. Patient confidentiality was ensured through strict data protection measures, including encrypted storage and limited access to authorized study personnel.

## 3. Results

This section presents the clinical characteristics and baseline data of the 52 patients who underwent autologous osteochondral transplantation (AOT) of the knee. The descriptive statistics summarize patient demographics, defect characteristics, and surgical details. These findings form the basis for the subsequent evaluation of clinical outcomes over time.

### 3.1. Patient Demographics and Baseline Characteristics

The cohort initially included 60 patients who underwent autologous osteochondral transplantation of the knee. Eight patients were excluded from the final analysis: six due to incomplete clinical data or due to loss of follow-up, one following a second trauma resulting in a meniscal lesion, and one due to revision surgery necessitated by an incomplete primary reconstruction. The final study population thus consisted of 52 patients with a mean age of 42.3 years (range: 14–74 years, SD ± 13.6) and a median age of 44 years. Of these, 26 patients (50.0%) were female and 26 (50.0%) were male. Laterality of the operated knee was predominantly right-sided (61.5%, *n* = 32), with 20 patients (38.5%) treated on the left side (Table 1).

Regarding osteochondral lesion severity, 24 patients (46.2%) were classified as ICRS grade III, while the other 28 patients (53.8%) presented with grade IV lesions. These baseline findings illustrate a diverse patient population, with a broad range of defect sizes and reconstruction complexities, providing a solid foundation for evaluating postoperative outcomes.

### 3.2. Surgical Outcomes

All procedures were performed by a single, high-volume orthopedic surgeon using a standardized technique. Osteochondral defects were prepared with a water-cooled diamond cutting system that enabled precise, wet-grinding of the recipient bed. Cylindrical osteochondral grafts were harvested from non-weight-bearing zones of the ipsilateral patellofemoral groove and press-fit into the defect. Donor sites were reconstructed with autologous bone cylinders from the iliac crest, covered with musculoskeletal tissue to promote healing; the iliac crest cavity was refilled with a hydroxyapatite-based substitute and the musculotendinous sheath was closed for stability.

The mean total transplant area was 196.6 ± 88.1 mm^2^ (range: 56.75–399.62 mm^2^). Graft location was most frequently the medial femoral condyle (47.1%), followed by the central patella (17.1%), lateral femoral condyle (15.7%), medial patellar groove (12.9%), and tibial plateau (7.1%).

Defects were reconstructed with one to four overlapping cylinders within a single compartment (mean: 1.8 cylinders ± 0.84). A given patient could receive grafts in up to three compartments; nearly half required a double-cylinder reconstruction in one compartment, whereas the remainder needed more complex configurations. Overall, patients received a mean of 2.47 cylinders (±0.85). Donor site harvesting involved the lateral patellar groove in 37.1% of knees, the medial groove in 2.9%, and both grooves in 60.0%, reflecting variable graft demand.

No major surgical complications occurred. There were no instances of intra-articular bleeding, persistent effusion, donor site knee pain, or infection, and no patient required conversion to arthroplasty during follow-up. One patient developed persistent hypoesthesia of the lateral cutaneous femoral nerve, attributed to an anatomical variant encountered at the iliac crest harvest site. No additional donor site morbidities were reported, underscoring the safety and reproducibility of the surgical approach.

### 3.3. Pain Reduction over Time (VAS Scores)

Pain intensity was assessed using the VAS at five postoperative timepoints: baseline (preoperative), 3 months, 6 months, 9 months, and 12 months. At baseline, the mean VAS score for the entire cohort (*n* = 52) was 6.32 (SD ± 2.37), indicating moderate-to-severe pain. A substantial reduction was observed at 3 months postoperatively (mean = 1.14, SD ± 1.40), which further decreased at 6 months (mean = 0.83, SD ± 0.85), 9 months (mean = 1.13, SD ± 1.62), and stabilized by 12 months (mean = 0.72, SD ± 0.92).

Generalized Estimating Equations (GEEs) confirmed a highly significant effect of time on VAS reduction (*p* < 0.001). Post hoc pairwise comparisons showed that the VAS scores at all postoperative timepoints were significantly lower than at baseline (all *p* < 0.001). No statistically significant differences were found between the 3-, 6-, 9-, and 12-month scores, indicating that the greatest improvement occurred within the first 3 months after surgery and was maintained thereafter.

**Figure 3:**
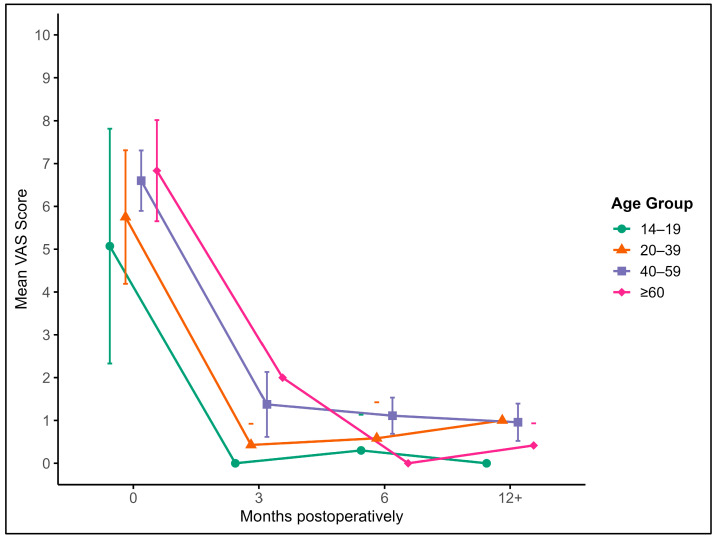
Mean VAS with 95% confidence intervals and outliers at baseline (0), 3, 6, 9, and 12+ months postoperatively, stratified by age groups: (green) 14–19 years, (orange) 20–39 years, (blue) 40–59 years, and (purple) ≥ 60 years.

Notably, when stratified by age category, all age groups experienced consistent postoperative pain reduction, though adolescents (14–19 years) appeared to report slightly lower baseline pain and a faster decline. Older adults (≥60 years) also showed meaningful improvements, though with slightly higher residual pain at 9 months compared to younger cohorts. These trends, while not statistically significant between groups, may reflect age-related differences in pain perception or recovery dynamics. Figure 1 illustrates the average VAS score and its 95% confidence interval across these intervals, stratified by age groups: adolescents (14–19 years), young adults (20–39 years), middle-aged adults (40–59 years), and older adults (60+ years) (Figure 3).

Across all age groups, a marked reduction in pain is observed within the first 3 months post-surgery, with sustained low pain levels thereafter. Younger patients tended to report slightly lower baseline pain and faster improvement, while older patients showed more gradual but consistent pain relief over time.

The multivariable model further revealed that pain levels were significantly influenced by the anatomical site of the graft (*p* < 0.001) and patient age (*p* = 0.006), while graft size and sex had no significant effects. Although correlation analyses showed trends toward higher pain scores in older patients at 3 and 6 months, none of these associations reached statistical significance (*p* > 0.05).

### 3.4. Functional Outcomes: Tegner–Lysholm, Modified Cincinnati, and Knee Society Scores

Postoperative knee function was assessed using the Tegner–Lysholm Knee Score (TLKS) and the Knee Society Score (KSS), both of which demonstrated significant and sustained improvements following autologous osteochondral transplantation.

#### 3.4.1. Tegner–Lysholm Knee Score (TLKS)

**Figure 4:**
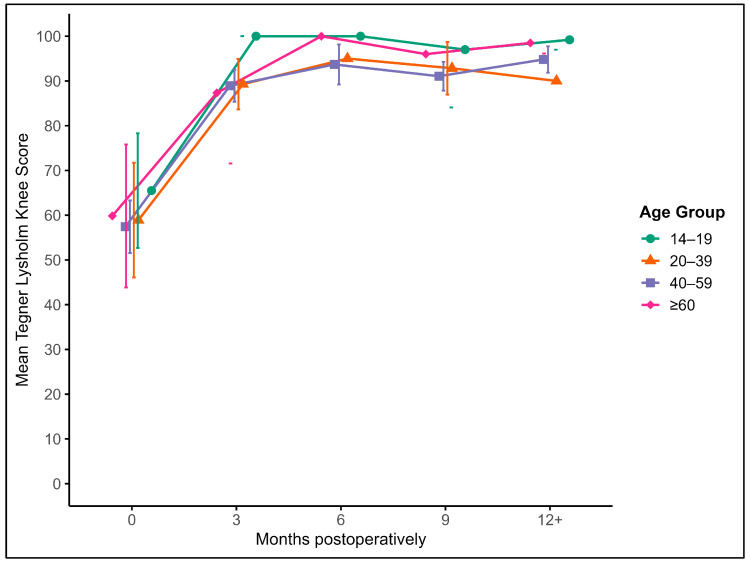
Mean Tegner–Lysholm Knee Scores with 95% confidence intervals and outliers at baseline and at 3, 6, 9, and 12+ months postoperatively, stratified by age groups: (green) 14–19 years, (orange) 20–39 years, (blue) 40–59 years, and (purple) ≥ 60 years.

Patients showed a marked increase in the TLKS from a preoperative mean of 58.6 to 89.5 at 3 months. Scores continued to improve over time, reaching 92.7 at 6 months, 94.6 at 9 months, and 95.0 at 12 months or later. These improvements were statistically significant over time (*p* < 0.001), with the largest gains occurring within the first three months post-surgery. Functional recovery remained stable beyond six months (Figure 4).

There were no significant correlations between the TLKS and patient age, sex, or graft size at any timepoint. Although graft localization showed a trend toward influencing outcomes, this did not reach statistical significance (*p* = 0.052).

#### 3.4.2. Knee Society Score (KSS)

**Figure 5:**
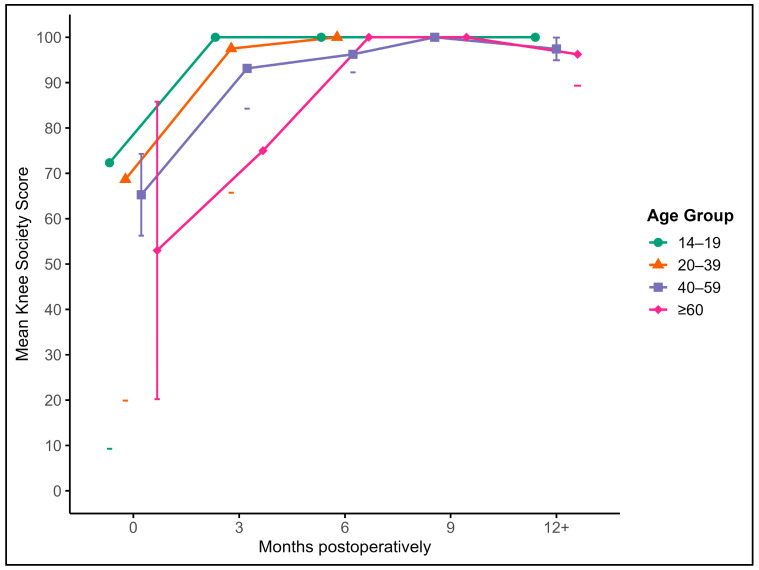
Mean Knee Society Scores (KSSs) with 95% confidence intervals and outliers at baseline and at 3, 6, 9, and 12+ months postoperatively, stratified by age groups: (green) 14–19 years, (orange) 20–39 years, (blue) 40–59 years, and (purple) ≥ 60 years.

A similar trend was observed with the KSS. The mean baseline score of 63.8 increased to 92.3 at 3 months and continued to improve to 96.6 at 6 months, 97.8 at 9 months, and 97.4 at 12 months or beyond. The effect of time on the KSS was significant (*p* < 0.001), and scores at all postoperative timepoints were significantly higher than at baseline. Improvements occurred early and remained consistent across the follow-up period (Figure 5).

While there was a modest inverse association between age and the final KSS, it did not reach significance. Graft size was significantly associated with a lower preoperative KSS (*p* = 0.011), but had no measurable influence on postoperative outcomes.

Together, the TLKS and KSS data reflect substantial and stable functional recovery following AOT, with the greatest improvements observed within the first few months postoperatively and sustained through long-term follow-up.

#### 3.4.3. Modified Cincinnati Score (MCS)

**Figure 6:**
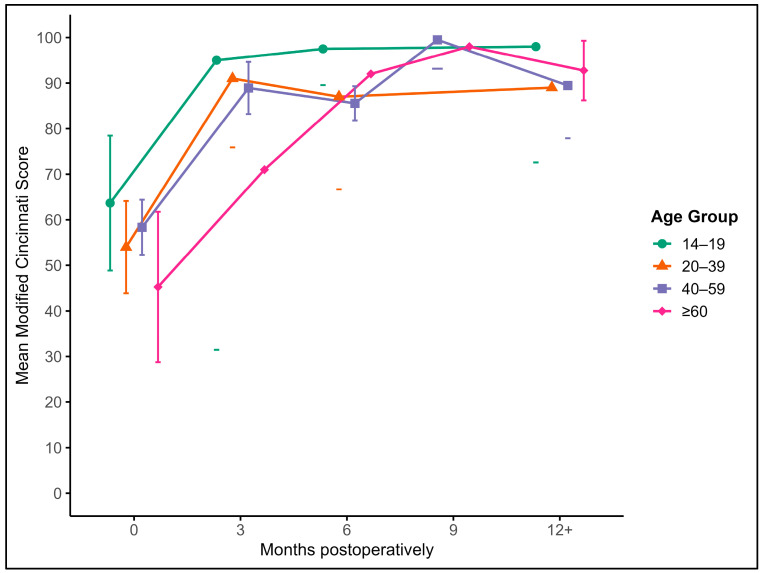
Mean Modified Cincinnati Scores with 95% confidence intervals and outliers at baseline and at 3, 6, 9, and 12+ months postoperatively, stratified by age groups: (green) 14–19 years, (orange) 20–39 years, (blue) 40–59 years, and (purple) ≥ 60 years.

Postoperative knee function as measured by the Modified Cincinnati Score showed a significant and sustained increase across all age groups. The mean preoperative score of 54.1 improved to 84.9 at 3 months, 90.5 at 6 months, 93.2 at 9 months, and 93.8 at 12 months or later. This improvement over time was statistically significant (*p* < 0.001), with most gains observed in the early postoperative phase. There were no significant correlations between Modified Cincinnati Scores and patient age, sex, or the number of transplanted cylinders. Functional outcomes remained high and stable beyond six months postoperatively (Figure 6).

### 3.5. Intergroup Analysis of Transplanted Cylinder Grafts

**Figure 7:**
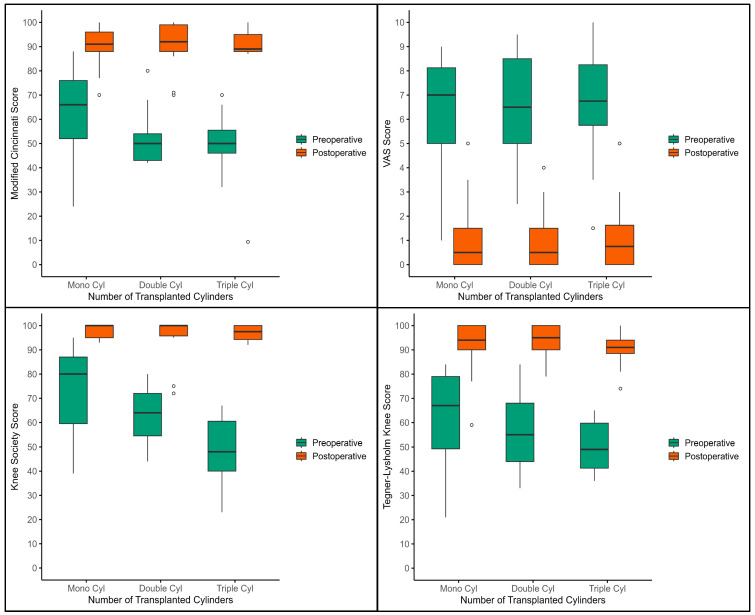
Box-and-whisker plots with outliers of preoperative versus pooled postoperative VAS, TLKS, KSS, and MCS, grouped by number of grafts (mono, double, triple). Boxes represent the inter-quartile range, horizontal lines the median, whiskers 1.5 × IQR, and points individual outliers. The figure illustrates the uniform, significant improvement across all groups and the absence of meaningful intergroup differences at follow-up.

Patients were stratified according to the number of overlapping grafts used to reconstruct a single defect: mono-, double-, triple-, and (in one case) quadruple-cylinder rows. Because only one patient received a quadruple reconstruction, this subgroup was excluded from formal statistical comparison (Figure 7).

Functional (TLKS, KSS, MCS) and pain (VAS) scores improved significantly from baseline to the final follow-up in all three analyzed groups (Wilcoxon signed-rank, *p* < 0.001 for every score). Intergroup comparisons of postoperative outcomes (Mann–Whitney U) showed no significant differences between mono-, double-, and triple-cylinder reconstructions (all *p* > 0.05).

Patients who required double or triple cylinders entered surgery with slightly worse function and higher pain—consistent with their more extensive lesions—yet achieved postoperative results that were statistically and clinically indistinguishable from those of the mono-cylinder cohort. These findings indicate that more complex, multi-cylinder reconstructions can deliver pain relief and functional recovery equivalent to those of simpler single-plug procedures.

## 4. Discussion

This study confirms the clinical value of autologous osteochondral transplantation (AOT) as a reliable technique for treating large osteochondral defects of the knee, resulting in stable mid-term improvements in both pain and knee function [26,27]. Our findings align with earlier studies showing that press-fit osteochondral grafts, when harvested and implanted under optimized surgical conditions, can restore joint surface congruity and maintain structural integrity over time [28,29]. We hypothesize that the robust outcomes in larger defects arise from reconstitution of the osteochondral unit: hyaline cartilage resurfacing, subchondral bone continuity, and press-fit stability (including overlapping plug configurations) reduce interfacial shear and rim overload, promote osteointegration, and help maintain chondral viability over time.

Compared to the pain scores reported in the literature, our results show similar or even better outcomes in patients undergoing AOT for large osteochondral knee lesions. The mean pain score in our cohort dropped from 6.1 before surgery to 1.5 one year after the procedure. Papakostidou et al. [30] reported a similar improvement, with the VAS scores decreasing from 8.7 to 1.6 in patients undergoing total knee arthroplasty. However, their study population was older and mostly affected by advanced osteoarthritis, whereas our cohort included a wider age range and focused on focal cartilage defects that often required technically demanding overlapping grafts.

A further comparison with the study by Mekkawy et al. [31] reveals important differences in postoperative pain development. In their prospective study on total knee arthroplasty, pain scores improved more slowly, from 5.8 immediately after surgery to 3.8 at six months. In contrast, our patients experienced a rapid and sustained decrease in pain, with the mean VAS dropping from 6.32 preoperatively to 1.14 after three months and stabilizing at 0.72 by one year. This faster recovery may be due to the biological preservation of native joint structures and the precision of the surgical technique. While pain in TKA patients persisted longer and was more diffuse in origin, our results suggest more localized and predictable symptom relief. The faster and more consistent pain reduction seen with AOT highlights its potential to offer better short- and mid-term pain outcomes in suitable patients.

When comparing functional outcomes, our results also indicate strong improvements relative to the published literature. In our cohort, the Tegner–Lysholm Knee Score improved from a baseline of 58.6 to 95.0 at the final follow-up, and the Knee Society Score rose from 63.8 to 97.4. These improvements occurred primarily within the first six months and were maintained over time. Papakostidou et al. [30] reported a KSS improvement from 45.2 to 85.4 in their arthroplasty group, while Kuroda et al. [32] observed a functional activity score increase from 43.0 to 65.8 and an objective KSS increase from 67.7 to 92.3. While all of these procedures led to meaningful improvements, our higher scores may reflect the advantages of joint preservation through AOT, particularly in younger or more active individuals.

No significant main-effect association between age and functional outcome scores at follow-up was observed. This lack of age effect may reflect selection of older patients with focal pathology amenable to AOT, uniformly standardized technique, and smaller mean defects in older subgroups. Therefore, age independence in this cohort likely reflects case selection and technical standardization that reduce biologic variability, rather than equivalence across all elderly patients.

The sustained improvement beyond six months across all age groups further emphasizes the long-term stability of AOT outcomes, an important consideration when comparing biological reconstruction with prosthetic solutions. Although no TKA control was available, in older patients meeting strict focal-lesion criteria, AOT may serve as a joint-preserving option that achieves early symptomatic relief and defers arthroplasty. Head-to-head comparative trials are warranted to define indications by age and joint status.

Compared to other joint-preserving surgical treatments for osteochondral defects of the knee, such as microfracture or autologous chondrocyte implantation, the outcomes reported in this study indicate superior improvements in both pain relief and knee function following AOT. Beris et al. [33] reported a VAS reduction from 6.5 to 2.0 and a TLKS increase from 53.2 to 89.3 at 24 months after autologous chondrocyte implantation, which demonstrates meaningful but slightly less pronounced improvement compared to our results (the VAS score dropped from 6.1 preoperatively to 1.5 at one year while the TLKS improved from 58.6 to 95.0). Similarly, Vasiliadis et al. [34] found that the VAS scores decreased from 7.1 to 2.3 and the TLKS increased from 50.2 to 87.8 following matrix-induced autologous chondrocyte implantation over a comparable follow-up period. These findings support the conclusion that AOT may lead to faster and more complete symptom relief and functional recovery, particularly in patients with larger lesions requiring stable and mechanically robust repair. The consistent and sustained improvements in our cohort suggest that AOT remains a valuable option in the context of biologic joint preservation, especially when technical demands such as overlapping graft placement can be met with precision.

Taken together, these findings support the use of autologous osteochondral transplantation as a safe and effective surgical treatment in younger or more active patients with focal cartilage damage greater than 200 mm^2^. AOT helps to keep the natural structure of the joint and allows normal movement, which may lead to faster recovery, better function, and less long-term pain. It also offers economic advantages. Since AOT does not use implants, it avoids many of the long-term problems and costs linked to prosthetic joints. In addition, the procedure can be repeated or adjusted later if needed, making it a good long-term option for preserving joint health [13,35,36].

While the results of this study are promising, several limitations should be considered. The retrospective study design may introduce selection or reporting bias, as data were based on clinical records and routine follow-up visits. Because no untreated or alternative-procedure control cohort was available, causal inference is limited. While the magnitude and early timing of improvement suggest a treatment effect, spontaneous or contextual recovery cannot be ruled out. Furthermore, this study did not include a control group, which limits the ability to directly compare AOT with other surgical techniques such as microfracture, autologous chondrocyte implantation, or total knee replacement. Comparisons with published studies are helpful but remain indirect due to differences in patient groups, methods, and surgical protocols.

Also, all operations were performed by one experienced orthopedic surgeon. This ensured a high level of standardization but may limit the generalizability of the findings to other surgeons or clinical settings. Studies involving multiple surgeons and centers would help to confirm if similar outcomes can be achieved in broader practice.

Future research should focus on prospective, multicenter studies with longer follow-up periods. These studies should aim to compare AOT directly with other treatment options, investigate patient satisfaction and recovery patterns, and explore the most effective graft techniques and rehabilitation protocols. This will help to better define the clinical role of AOT and guide patient selection in joint preservation surgery.

## 5. Conclusions

This study demonstrates that autologous osteochondral transplantation is a reliable and effective treatment for large osteochondral defects of the knee, particularly those greater than 200 mm^2^. In a cohort of patients with complex and technically demanding lesions, the procedure resulted in significant and sustained improvements in both pain and knee function over the first year of follow-up. Compared to outcomes reported for other surgical methods, AOT provided faster pain relief and higher functional scores, likely due to its ability to preserve native joint structures and biomechanics. The use of a standardized surgical technique and the absence of major complications further support the feasibility and safety of this approach in specialized settings. Although the findings are encouraging, additional prospective, controlled, and multicenter studies with longer follow-up are needed to confirm these results, evaluate long-term durability, and optimize patient selection. AOT may represent a valuable biological alternative to prosthetic joint replacement for focal cartilage lesions exceeding 200 m^2^, especially in younger or more active individuals.

## Figures and Tables

**Table 1:** Demographic and clinical characteristics by age group.

Category	Total (*n* = 52)	14–24 Years	25–39 Years	40–59 Years	60–74 Years
Sex, *n* (%):					
– Female	24 (46.2%)	2 (28.6%)	6 (66.7%)	12 (38.7%)	4 (80.0%)
– Male	28 (53.8%)	5 (71.4%)	3 (33.3%)	19 (61.3%)	1 (20.0%)
Side of Surgery, *n* (%):					
– Right Knee	33 (63.5%)	3 (42.9%)	7 (77.8%)	19 (61.3%)	4 (80.0%)
– Left Knee	19 (36.5%)	4 (57.1%)	2 (22.2%)	12 (38.7%)	1 (20.0%)
ICRS Grade, n (%):					
– Grade III	24 (46.2%)	4 (57.1%)	1 (11.1%)	14 (45.2%)	5 (100.0%)
– Grade IV	28 (53.8%)	3 (42.9%)	8 (88.9%)	17 (54.8%)	0 (0.0%)
Total Graft Size (mm^2^):					
– Mean (SD)	224.4 (±84.5)	271.7	221.5	224.0	165.9
– Range	71.6–399.6	110.3–399.6	129.7–312.2	71.6–382.5	110.3–239.7

## Data Availability

The data presented in this study are available upon request from the corresponding author.

## Abbreviations

The following abbreviations are used in this manuscript:

AOT	Autologous Osteochondral Transplantation
CI	Confidence Interval
DBCS	Diamond Bone Cutting System
DRG	Diagnosis-Related Groups
GEE	Generalized Estimating Equation
ICRS	International Cartilage Repair Society
KSS	Knee Society Score
LSD	Least Significant Difference
MCS	Modified Cincinnati Score
OD	Osteochondral Defect
SD	Standard Deviation
SPSS	Statistical Package for the Social Sciences
TLS	Tegner–Lysholm Score
VAS	Visual Analog Scale
